# The Preparation and CO_2_-Resistant Performance of a Smart Responsive Polymer Gel for CO_2_ Flooding Channel Blocking

**DOI:** 10.3390/molecules31030514

**Published:** 2026-02-02

**Authors:** Xiangjuan Meng, Mingwei Zhao, Zhenfeng Ma, Xinjie Xu, Zhongzheng Xu, Yuxin Xie, Yining Wu, Ziyi Wang, Wenhao Ren, Huan Zhang

**Affiliations:** 1 State Key Laboratory of Deep Oil and Gas, China University of Petroleum (East China), Qingdao 266580, China; mengxj-tlm@petrochina.com.cn (X.M.); xuxinjie1028@163.com (X.X.); upc_xzz@163.com (Z.X.); xieyx3655@163.com (Y.X.); wuyining@126.com (Y.W.); 19511641057@163.com (Z.W.); 18509900604@163.com (W.R.); 18330840841@163.com (H.Z.); 2 Shandong Key Laboratory of Oil and Gas Field Chemistry, China University of Petroleum (East China), Qingdao 266580, China

**Keywords:** polymer gel, smart responsive, channel blocking, CO_2_-resistant performance

## Abstract

CO_2_ flooding is an effective technique for enhancing oil recovery in low-permeability reservoirs. However, it is often hindered by severe CO_2_ channeling. This challenge is particularly pronounced in near-wellbore regions with large pressure differentials and in fractured reservoirs, where high CO_2_ injection rates and rapid breakthrough require channel blocking systems with high mechanical strength and excellent CO_2_-resistant performance. In this work, a smart responsive polymer was synthesized and subsequently crosslinked with a highly active phenolic resin crosslinking agent to develop a smart responsive polymer gel channel blocking system. The resulting gel exhibits CO_2_-responsive strength enhancement and excellent CO_2_-resistant performance. The static and dynamic gelation behaviors, nonlinear rheological properties, CO_2_-resistant performance, channel blocking, and enhanced oil recovery performance of the smart responsive polymer gel were systematically investigated. The results demonstrate that the polymer gel maintains good structural stability during dynamic transport in the reservoir and does not undergo significant strength degradation under shear conditions. Moreover, the smart responsive polymer gel exhibits excellent CO_2_-resistant performance within a temperature range of 80–110 °C, salinity up to 10 × 10^4^ mg/L, and pressure up to 20 MPa. Moreover, the system shows a significant enhancement in channel blocking and enhanced oil recovery performance, highlighting its promising potential for effective CO_2_ flooding channel blocking in low-permeability reservoirs.

## 1. Introduction

At present, most conventional oil reservoirs have entered the late stage of development, making low-permeability reservoirs an important source of replacement energy [1,2]. However, low-permeability reservoirs are characterized by poor reservoir properties, low porosity and permeability, strong heterogeneity between the matrix and fractures, and complex pore structures, which greatly increase the difficulty of efficient exploitation [3,4,5]. Currently, the development of low-permeability reservoirs mainly relies on the injection of water, gas, or other fluids to supplement reservoir energy and enhance oil recovery [6,7]. Compared with water flooding, gas injection can more readily access micro- and nanopores, thereby more effectively displacing the residual oil in the reservoir matrix and improving overall recovery [8].

CO_2_ is a major component of greenhouse gases and is also widely recognized as an effective agent for supplementing reservoir energy and enhancing oil production. CO_2_ flooding technology involves injecting CO_2_ into oil reservoirs, which simultaneously enhances oil recovery and enables subsurface CO_2_ storage, offering the dual benefits of increased oil production and the achievement of carbon neutrality goals [9,10,11]. During CO_2_ flooding, the dissolution of CO_2_ in crude oil leads to oil swelling, viscosity reduction, and interfacial tension lowering, all of which significantly improve oil displacement recovery [12,13,14]. However, compared with reservoir crude oil, CO_2_ has a much lower viscosity and density. Under heterogeneous reservoir conditions, this disparity often results in viscous fingering and gravity segregation during the displacement process, thereby reducing sweep efficiency [15]. Moreover, many oil reservoirs that are suitable for CO_2_ flooding are commonly subjected to reservoir stimulation to achieve higher productivity, leading to the development of artificial fractures [16,17]. Due to the pronounced contrast in petrophysical properties between the matrix and fracture systems, injected CO_2_ preferentially channels through fractures, causing early breakthrough and a significant reduction in CO_2_ flooding oil recovery [18,19]. Therefore, addressing CO_2_ channeling during CO_2_ flooding is critical for improving oil recovery.

Unlike conventional waterflooding channeling, CO_2_ under reservoir conditions typically exists in a supercritical state, characterized by extremely low viscosity and strong mobility in pores and fractures [20,21]. In addition, the high injection intensity during CO_2_ flooding further exacerbates channeling, thereby imposing more stringent requirements on the strength, stability, and adaptability of channel blocking systems, particularly in near-wellbore regions with high pressure differentials and within fracture networks. High-strength and continuous polymer gel channel blocking systems have demonstrated superior blocking performance, effectively suppressing CO_2_ channeling and improving displacement recovery [22,23,24]. Consequently, polymer gel systems are considered among the most promising technologies for CO_2_ channeling control. To date, considerable progress has been made in improving the reservoir adaptability of polymer gels. By modifying polymer backbones, the network strength and hydrophilicity of polymer gels can be enhanced, thereby improving their stability under high-temperature and high-salinity conditions [25,26]. He et al. [27] developed a polymer particle composite gel temporary plugging agent (PCGTPA) with a density ranging from 1.0 to 1.15 g/cm^3^ and an operating temperature window of 85–165 °C. Temporary plugging tests demonstrated that PCGTPA exhibits excellent pressure bearing capacity, gas channeling prevention, and minimal formation damage. Zhang et al. [28] designed a thixotropic polymer gel system through molecular design and compositional optimization. Compared with polymer solutions, this system exhibited a larger hysteresis area, indicating favorable thixotropic behavior prior to gelation, and achieved complete gelation within the temperature range of 80–140 °C. At 120 °C, the gel showed a viscosity of 7500 mPa·s, an elastic modulus of 51 Pa, and a viscous modulus of 6 Pa, demonstrating good shear thixotropy. Despite these advances, existing polymer gel channel blocking systems still face significant challenges. CO_2_ can dissolve in formation water or directly diffuse into the gel matrix, creating an acidic environment that induces degradation and volumetric shrinkage of conventional polymer gels, thereby significantly reducing their channel blocking performance.

In this work, a smart responsive polymer was synthesized and subsequently crosslinked with a highly active phenolic resin crosslinking agent to develop a smart responsive polymer gel channel blocking system. The static and dynamic gelation behaviors, nonlinear rheological properties, CO_2_-resistant performance, channeling blocking and enhanced oil recovery performance of the smart responsive polymer gel were systematically investigated. These results elucidate the CO_2_-resistant and applicable operating window of the polymer gel, providing a theoretical basis and practical technical guidance for its field application.

## 2. Results and Discussion

**Figure 1:**
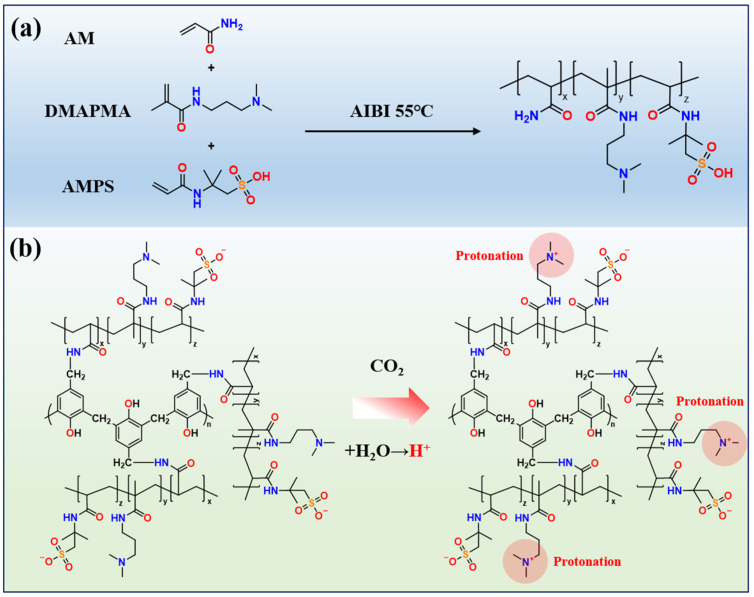
(**a**) The synthetic structure of the CO_2_-responsive polymer. (**b**) The CO_2_ response and CO_2_ resistance mechanism of the smart responsive polymer gel.

The synthetic structure of the CO_2_-responsive polymer is shown in Figure 1a. It was prepared via solution polymerization of the monomers AM, DMAPMA, and AMPS, which provide crosslinkable groups, temperature- and salt-resistant groups, and protonatable functional groups. The CO_2_ response and CO_2_ resistance mechanism of the smart responsive polymer gel is illustrated in Figure 1b. When CO_2_ dissolves in water, it reacts with water molecules and dissociates to release H^+^. The smart responsive polymer gel contains abundant quaternary amine groups, which become protonated under acidic conditions. This protonation enhances the hydrophilicity of the polymer chains and increases the electrostatic repulsion between them. Such effects not only significantly improve the mechanical strength of the gel, but also promote the formation of the more stable three-dimensional network structure at the microscopic scale. The strengthened interactions between the polymer network and water molecules further enhance the water retention capacity of the gel. The degradation mechanism of polymer gels in CO_2_ environments is mainly associated with the acceleration of hydrolysis at crosslinking sites (amide groups) by H^+^, which weakens the gel network structure. In contrast, in the smart responsive polymer gel, the protonation of quaternary amine groups imparts strong positive charges to the side chains. These positively charged side chains can effectively shield the crosslinking sites from H^+^ attack, thereby reducing hydrolysis-induced network loosening and preventing the associated loss of mechanical strength.

### 2.1. Static and Dynamic Gelation Properties of the Polymer Gel

**Figure 2:**
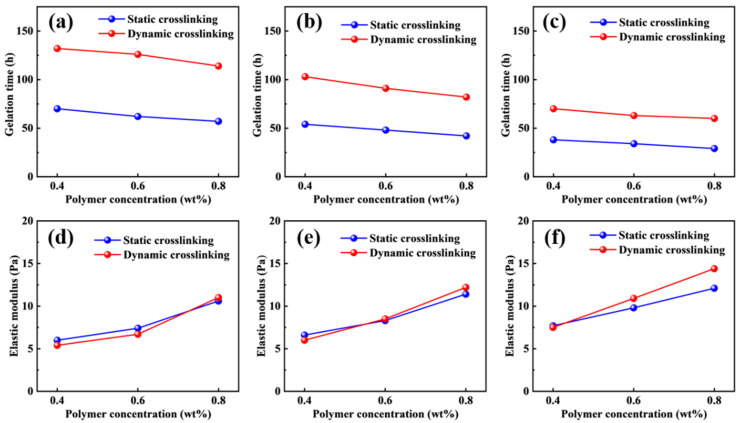
Static and dynamic gelation time of the smart responsive polymer gel at crosslinking agent concentrations of 0.8 wt% (**a**), 1.2 wt% (**b**), and 1.6 wt% (**c**). Static and dynamic crosslinking elastic modulus of the smart responsive polymer gel at crosslinking agent concentrations of 0.8 wt% (**d**), 1.2 wt% (**e**), and 1.6 wt% (**f**).

The static and dynamic gelation time of different polymer gel formulations at 110 °C are presented in Figure 2a–c. Under identical formulation conditions, the gelation time under dynamic conditions is consistently longer than that under static conditions, indicating that flow-induced shear significantly retards the gelation process of the smart responsive polymer gel. This trend is observed across all investigated crosslinking agent and polymer concentrations, demonstrating that the shear environment plays a critical role in governing the gelation kinetics of the polymer gel system.

Compared with polymer concentration, crosslinking agent concentration exerts a more pronounced influence on the gelation time. As the crosslinking agent concentration increases, both static and dynamic gelation time exhibit a gradual decrease, and this reduction is more significant under dynamic conditions. This behavior can be attributed to the fact that the crosslinking reaction of polymer gels inherently depends on the effective dispersion and interaction of crosslinking agent molecules in the vicinity of the amide groups along the polymer chains. Under flowing conditions, continuous shear induces repeated redistribution and migration of the crosslinking agent, thereby reducing the probability of effective collisions between crosslinking agent molecules and active sites on the polymer chains. As a result, the frequency of crosslinking events decreases, leading to a reduction in crosslinking efficiency. Furthermore, the formation of a three-dimensional polymer gel network requires a relatively stable reaction environment to allow crosslinking points to develop progressively and interconnect, ultimately forming a continuous spatial network. Under dynamic flow conditions, shear disturbances continuously disrupt the evolving gel structure and may partially break already formed local crosslinked domains, thereby inhibiting the continuous growth of the crosslinked network and further delaying the overall gelation process. Consequently, the gelation time of the polymer gel is significantly prolonged under dynamic conditions and exhibits enhanced sensitivity to variations in crosslinking agent concentration.

The elastic modulus of the polymer gel formed under static and dynamic crosslinking conditions at 110 °C is presented in Figure 2d–f. Under identical formulation conditions, the elastic modulus of the polymer gel obtained under dynamic gelation is generally comparable to that formed under static conditions, and it increases progressively with increasing polymer and crosslinking agent concentrations. These results indicate that the flow-induced shear environment does not exert a significant adverse effect on the mechanical strength of the final polymer gel network. This demonstrates that the smart responsive polymer gel system exhibits good structural stability during dynamic transport within the reservoir and does not undergo pronounced strength degradation as a result of shear effects.

During injection, a sufficiently delayed gelation process allows the gel-forming solution to maintain relatively low flow resistance prior to complete crosslinking, thereby facilitating deeper penetration into the formation and preferential filling of high-permeability channels [29]. Accordingly, the gelation and blocking time of the system can be tailored by adjusting the polymer concentration, crosslinking agent concentration, and their mixing ratio, enabling flexible adaptation to different operational scenarios. For near-wellbore regions that require rapid blocking, a shorter gelation time may be preferable. In contrast, for applications involving deeper profile control that require extended transport distances, a longer gelation time is more suitable. Upon reaching the target blocking zone, the system is still capable of forming the gel structure with high elastic modulus and mechanical strength, thereby effectively achieving profile control and channel blocking functions.

### 2.2. Nonlinear Rheological Properties of the Polymer Gel

**Figure 3:**
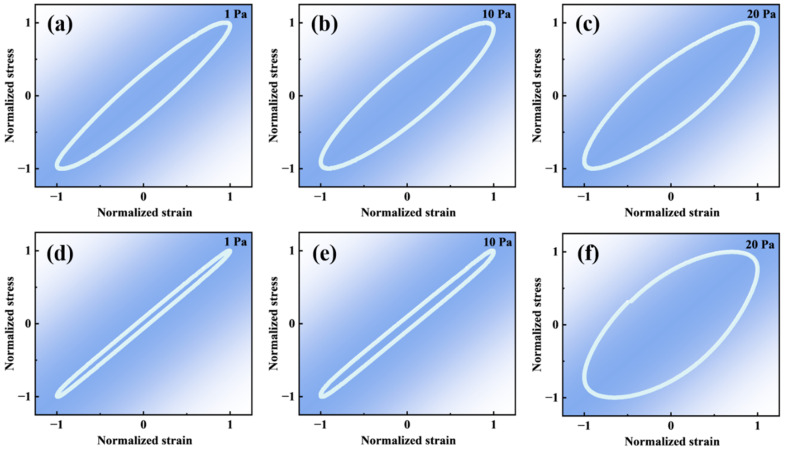
Elastic Lissajous curves of the polymer gel before CO_2_ response under stress amplitudes of 1 Pa (**a**), 10 Pa (**b**), and 20 Pa (**c**). Elastic Lissajous curves of the polymer gel after CO_2_ response under stress amplitudes of 1 Pa (**d**), 10 Pa (**e**), and 20 Pa (**f**).

The elastic characteristics of polymer gels determine their ability to resist deformation and structural failure under external stress and constitute one of the key parameters governing their channel blocking performance and long-term stability. To further elucidate the nonlinear elastic behavior of the polymer gel before and after CO_2_ response, elastic Lissajous curves were measured at three stress amplitudes of 1 Pa, 10 Pa, and 20 Pa, as shown in Figure 3. The elastic Lissajous curves of the polymer gel under different stress conditions generally exhibit elongated elliptical shapes with varying minor axes. For elastic Lissajous representations, the enclosed area reflects the energy dissipation of the material under cyclic deformation, whereas the length of the minor axis is closely associated with the viscous contribution of the system. A shorter minor axis indicates a more elasticity-dominated response with weaker viscous flow behavior, implying a lower tendency for irreversible deformation under applied stress [30].

Under low-stress conditions (1 Pa), the polymer gel before and after CO_2_ response remains within the linear viscoelastic regime, and the corresponding elastic Lissajous curves display highly elongated elliptical shapes, indicating that elastic behavior predominates and the gel network structure remains intact. The material response under these conditions is characterized mainly by reversible deformation. A comparison between the states before and after the response reveals that the polymer gel after CO_2_ response exhibits a further reduction in the minor axis of the elastic Lissajous curve, suggesting a higher elastic contribution relative to the sample before response. This observation indicates that the CO_2_ response process enhances the elastic characteristics of the polymer gel network to a certain extent.

With increasing stress amplitude (10 Pa and 20 Pa), the system gradually transitions from the linear viscoelastic regime to the nonlinear viscoelastic regime. Nevertheless, even at higher stress levels, the elastic Lissajous curves of the polymer gel before and after CO_2_ response do not exhibit pronounced distortion or deviation from an elliptical shape. This behavior demonstrates that the polymer gel maintains favorable elastic response characteristics over a broad stress range, indicating that the three-dimensional crosslinked network formed within the polymer gel possesses high structural stability and can effectively withstand substantial external perturbations without significant structural degradation.

### 2.3. CO_2_-Resistant Performance of the Polymer Gel at Different Temperatures

**Figure 4:**
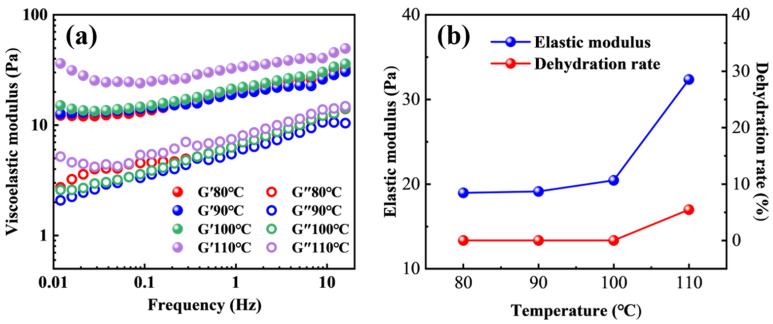
The effect of temperature on the viscoelastic modulus (**a**) and dehydration rate (**b**) of the polymer gel.

During CO_2_ flooding operations, the high-temperature and high-pressure conditions of the reservoir environment can significantly affect the physical structure and chemical stability of polymer gels, thereby limiting their long-term stability and channel blocking effectiveness under CO_2_ conditions [31]. Therefore, a systematic investigation into the effect of temperature on the performance of the smart responsive polymer gel is of considerable theoretical significance and practical relevance. Figure 4 illustrates the variation in the viscoelastic modulus of the polymer gel under different temperatures in a CO_2_ environment at 20 MPa. The elastic modulus (G′) of the polymer gel exhibits a pronounced increasing trend with increasing temperature under CO_2_ conditions. Within the temperature range of 80–110 °C, the polymer gel consistently displays elastic-dominated behavior, characterized by an elastic modulus exceeding the viscous modulus (G″), indicating that a relatively stable gel structure is maintained even under high temperature and CO_2_ conditions. Quantitative analysis reveals that when the temperature increases from 80 °C to 110 °C, the elastic modulus of the polymer gel increases by 70.62%, demonstrating a significant temperature-strengthening effect.

This pronounced enhancement in elastic modulus can be attributed to the synergistic contributions of several factors. First, elevated temperature partially suppresses the detrimental effects of CO_2_ on the polymer gel network, mitigating the adverse impact of the acidic CO_2_ environment on the polymer backbone and crosslinked structure. Second, under the weakly acidic conditions induced by CO_2_, electrostatic interactions and supramolecular associations among polymer chains are strengthened, facilitating the formation of a denser physical crosslinking network and thereby enhancing the overall network strength of the polymer gel. In addition, the reactivity of the highly active phenolic resin crosslinking agent is significantly enhanced at elevated temperatures, promoting more complete chemical crosslinking reactions and further improving the stability and mechanical strength of the three-dimensional polymer gel network [32].

Figure 4b presents the variations in dehydration rate of the polymer gel at different temperatures. It can be observed that within the temperature range of 80–110 °C, the polymer gel maintains good structural stability, with the dehydration rate remaining at a relatively low level. Even at 110 °C, the dehydration rate is only 5.46%, indicating that the smart responsive polymer gel possesses excellent thermal resistance and dehydration resistance under high temperature and CO_2_ conditions. Consequently, the polymer gel is capable of maintaining stable structure and effective channel blocking performance over extended periods in complex reservoir environments, demonstrating promising potential for field applications.

### 2.4. CO_2_-Resistant Performance of the Polymer Gel at Different Salinities

**Figure 5:**
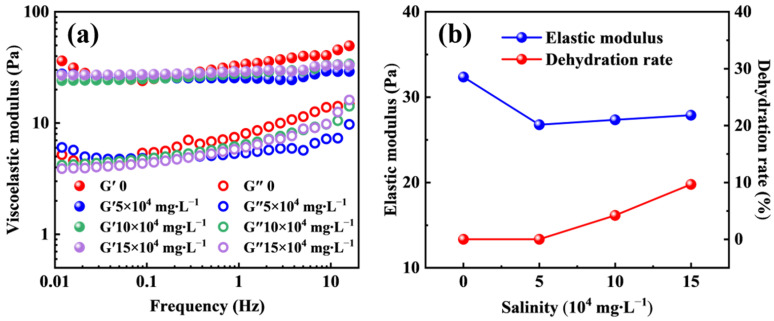
The effect of salinity on the viscoelastic modulus (**a**) and dehydration rate (**b**) of the polymer gel.

During CO_2_ flooding operations, in addition to temperature and pressure, the salinity of reservoir fluids also exerts a significant influence on the physical structural stability and chemical properties of polymer gels, thereby affecting their long-term stability and channel blocking performance under high-salinity CO_2_ environments. Figure 5 presents the variation in the viscoelastic modulus of the smart responsive polymer gel under different salinity conditions at 110 °C and 20 MPa CO_2_. As the salinity increases, the elastic modulus of the polymer gel exhibits an overall trend of an initial decrease followed by a slight increase. Under deionized water conditions, the polymer gel displays a relatively high elastic modulus of 32.35 Pa. When the salinity increases to 5 × 10^4^ mg/L, the elastic modulus decreases to 26.76 Pa, indicating that dissolved salt ions partially weaken the polymer gel network structure. With further increases in salinity, the variation in elastic modulus becomes less pronounced and tends to stabilize, suggesting that the polymer gel system is capable of maintaining relatively high structural strength even under high-salinity conditions.

As shown in Figure 5b, the dehydration rate of the polymer gel increases with increasing salinity. When the salinity reaches 15 × 10^4^ mg/L, the dehydration rate rises to 9.65%, indicating that a high-salinity environment significantly affects the water-retention capability of the polymer gel. This behavior is mainly attributed to strong electrostatic interactions between high concentrations of salt ions and hydrophilic functional groups on the polymer chains, such as carboxyl and sulfonate groups. These interactions shield the charges of the hydrophilic groups, weaken their hydration capacity, and consequently lead to the gradual loss of bound water and dehydration of the polymer gel.

The dehydration process induces shrinkage of the three-dimensional polymer gel network, resulting in an increased effective crosslink density per unit volume. This structural contraction partially offsets the adverse effect of increased salinity on the elastic modulus and may even lead to a slight recovery in elastic modulus at high salinity levels. However, the overall volume reduction of the polymer gel may impair its ability to effectively fill high-permeability channels or fractures, thereby reducing its channel blocking efficiency and being unfavorable for achieving long-term and stable conformance control.

### 2.5. CO_2_-Resistant Performance of the Polymer Gel at Different Pressures

**Figure 6:**
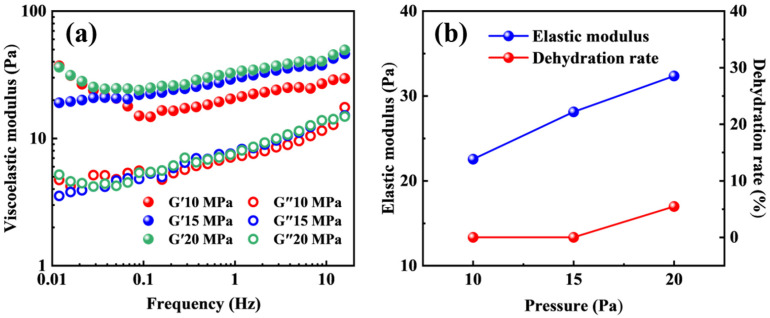
The effect of pressure on the viscoelastic modulus (**a**) and dehydration rate (**b**) of the polymer gel.

At a constant temperature of 110 °C, the effect of pressure on the performance of the polymer gel was systematically investigated at pressures of 10 MPa, 15 MPa, and 20 MPa. The variations in the viscoelastic modulus and dehydration rate of the polymer gel under different pressure conditions are presented in Figure 6. With increasing pressure, the elastic modulus of the polymer gel exhibits an overall slight upward trend, indicating that elevated pressure does not impose a pronounced adverse effect on the gel structure but instead enhances its mechanical properties to a certain extent. This behavior is primarily associated with the dissolution of CO_2_ under high-pressure conditions. As pressure increases, a greater amount of CO_2_ dissolves into the aqueous phase, where it reacts with water molecules and releases H^+^, thereby generating a weakly acidic environment. The smart responsive polymer gel system contains abundant tertiary amine groups, which are readily protonated under acidic conditions, causing the gel to transition from a nonionic state to a cationic state. Protonation of the tertiary amine groups markedly enhances the hydrophilicity of the polymer gel and increases electrostatic repulsion among polymer chains, leading to partial expansion of the three-dimensional network structure. During this process, more water molecules are absorbed and retained within the gel network pores, thereby improving the water-holding capacity and network flexibility of the polymer gel. The structural reinforcement induced by CO_2_ dissolution and the associated responsive behavior outweighs the potential detrimental effects of acidic conditions on polymer chain degradation or crosslinked network disruption. Consequently, the elastic modulus of the polymer gel exhibits a modest increase with increasing pressure.

As shown in Figure 6b, no obvious dehydration is observed for the polymer gel at pressures of 10 and 15 MPa, indicating good structural stability and water-retention capability under CO_2_ conditions. When the pressure increases to 20 MPa, slight dehydration of the gel is observed, with a dehydration rate of only 5.46%. This value remains relatively low and does not cause significant deterioration of the mechanical properties or structural integrity of the polymer gel.

### 2.6. Long-Term CO_2_-Resistant Performance of the Polymer Gel

**Figure 7:**
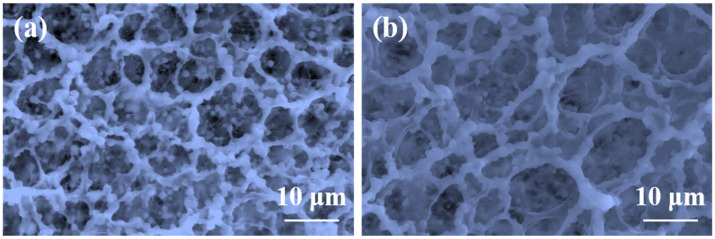
Microscopic morphology of the polymer gel before (**a**) and after (**b**) aging.

To evaluate the long-term CO_2_-resistant performance of the polymer gel, the microscopic morphology of the gel before and after 30 d of aging in a CO_2_ environment was examined, as shown in Figure 7. Prior to aging, the polymer gel exhibited a typical continuous porous three-dimensional network structure, characterized by uniformly distributed pores, well-defined pore walls, and good interconnectivity, indicating high structural integrity and spatial support capability. This uniform and compact network structure provides the gel with excellent elasticity and mechanical strength, forming the structural basis for its effective channel blocking performance and stability. After 30 d of aging, although certain changes in the microscopic pore morphology were observed, the gel still maintained a clearly interconnected three-dimensional network, demonstrating that the polymer gel retains good structural stability under prolonged CO_2_ aging conditions.

### 2.7. Channel Blocking and Enhanced Oil Recovery Performance

**Figure 8:**
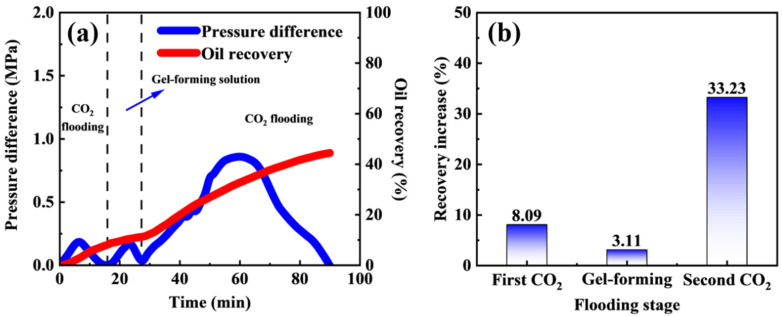
(**a**) Pressure difference and oil recovery curves during fractured core oil displacement experiment. (**b**) Oil recovery increase at different flooding stages.

The pressure difference and oil recovery results of the fractured core oil displacement experiment are shown in Figure 8. To more clearly illustrate the experiment results during the injection stage, the aging and gelation period was omitted. During the first CO_2_ flooding stage, the pressure difference increased slightly and then rapidly declined, with an oil recovery increase of 8.09%. This behavior is attributed to the presence of fracture in the core, through which CO_2_ preferentially channeled, primarily displacing a small amount of oil from the fracture while leaving a large proportion of the residual oil in the matrix. During the injection of the gel-forming solution, the displacement pressure difference also exhibited a slight increase followed by a rapid decrease, owing to the higher viscosity of the gel-forming solution compared with CO_2_. Meanwhile, oil recovery continued to increase, with a recovery increase of 3.11%. This improvement is mainly ascribed to the enhanced mobility ratio between the displacing fluid and oil, which effectively expanded the swept volume. After gelation, a second CO_2_ flooding stage was conducted. The displacement pressure difference underwent a rapid increase, followed by a stabilization stage and a subsequent rapid decline, and the recovery increase reached 33.23%. This substantial enhancement is attributed to the effective channel blocking of fracture by the polymer gel, which diverted more CO_2_ into the core matrix and enabled the displacement of residual oil trapped in the matrix [33,34]. These results demonstrate that the smart responsive polymer gel can significantly improve the sweep efficiency of CO_2_ flooding, enhance matrix oil mobilization, and thereby increase the overall CO_2_ flooding potential.

## 3. Materials and Methods

### 3.1. Materials

Acrylamide (AM, 99.9 wt%), dimethylaminopropyl methacrylamide (DMAPMA, 99 wt%), 2-acrylamido-2-methyl-1-propanesulfonic acid (AMPS, 98 wt%), and 2,2′-[azobis(1-methylethylidene)]bis[4,5-dihydro-1H-imidazole] dihydrochloride (AIBI, 96 wt%) were purchased from Shanghai Macklin Biochemical Co., Ltd., Shanghai, China. Hydrochloric acid (37 wt%) and sodium hydroxide (98 wt%) were obtained from Shanghai Aladdin Biochemical Technology Co., Ltd., Shanghai, China. The highly active phenolic resin crosslinking agent was supplied by Shandong Lifeng Chemical Co., Ltd., Jinan, China. All chemicals were used as received without further purification. Deionized water was prepared in the laboratory and used throughout the experiments. Crude oil was obtained from Tarim Oilfield. Artificial sandstone cores were supplied by Haian Oil Scientific Research Instruments Co., Ltd., Haian, China.

### 3.2. Synthesis of the Smart Responsive Polymer

The smart responsive polymer was synthesized via solution polymerization [35]. The detailed synthesis procedure is described as follows. First, 24.75 g of AM, 1.5 g of DMAPMA, and 3.75 g of AMPS were accurately weighed according to the predetermined formulation and sequentially added to 69 g of deionized water. The mixture was magnetically stirred at room temperature until all components were completely dissolved, yielding a homogeneous and transparent reaction solution. Subsequently, the pH of the solution was adjusted to 7.0 using dilute hydrochloric acid and sodium hydroxide solutions to minimize any adverse effects of acidic or alkaline conditions on the polymerization process and polymer structure.

The prepared reaction solution was then transferred to a three-necked flask and heated to 60 °C under continuous stirring. To eliminate the inhibitory effect of dissolved oxygen on polymerization, high-purity nitrogen was continuously purged through the system during the heating process, ensuring that the reaction proceeded under an inert atmosphere. Once the reaction temperature stabilized, 1.0 g of the initiator AIBI was added to initiate the polymerization. The reaction was allowed to proceed at 60 °C until completion. After completion of the polymerization, the resulting product was granulated and subsequently dried in a vacuum oven to remove residual water and unreacted species, ultimately yielding the smart responsive polymer for subsequent experiments.

### 3.3. Preparation of the Smart Responsive Polymer Gel System

A total of 0.6 g of the smart responsive polymer was weighed according to the predetermined formulation and added to 98.2 g of deionized water. The mixture was subjected to continuous magnetic stirring at room temperature until a homogeneous and transparent polymer solution was obtained. Subsequently, 1.2 g of the highly active phenolic resin crosslinking agent was added to the polymer solution and mixed thoroughly under continuous stirring to ensure uniform dispersion. The resulting reaction solution was then transferred into sealed ampoules and placed in a constant-temperature oven at 110 °C for 2 d to allow the gelation reaction to proceed. After completion of the crosslinking process, the smart responsive polymer gel was obtained.

### 3.4. Static and Dynamic Gelation Property Experiments

Based on the investigation of static gelation behavior, dynamic gelation experiments were further conducted to simulate the actual transport and gelation processes of the gel-forming solution under reservoir conditions. By introducing flow-induced shear, the gelation time and elastic modulus of the system under dynamic conditions were systematically evaluated. A high-temperature vortex oscillator was employed to simulate the continuous shear experienced by the gel-forming solution during migration through reservoir pores and fractures. The mass fraction of the smart responsive polymer was set to 0.4 wt%, 0.6 wt%, and 0.8 wt%, while the concentration of the highly active phenolic resin crosslinking agent was adjusted to 0.8 wt%, 1.2 wt%, and 1.6 wt%, respectively. All gelation experiments were carried out at 110 °C.

After gelation, the viscoelastic properties of the obtained polymer gels were characterized using a HAAKE MARS 60 rheometer (Thermo Fisher Scientiffc, Waltham, MA, USA). Prior to measurement, stress tests were performed to determine the linear viscoelastic region of the system. Subsequently, appropriate stress amplitudes and oscillation frequencies were selected, and a fixed amount of polymer gel samples was loaded into a concentric cylinder geometry to measure the elastic modulus. All rheological measurements were conducted at 25 °C.

### 3.5. Nonlinear Rheological Property Analysis

Stress scanning experiments were conducted to systematically evaluate the nonlinear viscoelastic behavior of the smart responsive polymer gel before and after CO_2_ response. During the experiments, large-amplitude sinusoidal shear stresses were applied, and the corresponding strain responses were recorded to analyze the nonlinear rheological characteristics of the polymer gel [36,37]. The onset of the nonlinear region was identified by monitoring the variations in the elastic modulus and viscous modulus. Subsequently, a representative stress level within the nonlinear region was selected for detailed rheological analysis. In this specific stress condition, elastic Lissajous curves were employed to characterize the viscoelastic behavior of the polymer gel in the nonlinear regime. To eliminate the influence of experimental conditions and facilitate comparison, all Lissajous curves were normalized. Stress amplitudes of 1 Pa, 10 Pa, and 20 Pa were selected for the nonlinear rheological measurements.

### 3.6. CO_2_-Resistant Performance Experiments

Under CO_2_ flooding conditions, reservoir parameters such as temperature, salinity, and pressure can significantly affect the physical structure and chemical stability of polymer gels, thereby constraining their long-term stability and channel blocking performance in CO_2_ environments. To systematically evaluate the CO_2_-resistant and applicable operating window of the smart responsive polymer gel, the effects of CO_2_ under different temperature, salinity, and pressure conditions on the gel performance were investigated. Specifically, the experimental temperatures were set at 80, 90, 100, and 110 °C, the salinity levels were 0, 5 × 10^4^, 10 × 10^4^, and 15 × 10^4^ mg/L, and the pressure conditions were 10, 15, and 20 MPa. Under these various CO_2_ environmental parameter combinations, the smart responsive polymer gel was subjected to aging treatments, and the variations in viscoelastic moduli and dehydration rate were measured to characterize the structural stability and dehydration resistance of the gel. For all experiments, the aging duration of the samples under the corresponding CO_2_ conditions was fixed at 10 d. In addition, a Sigma 300 scanning electron microscope (Zeiss, Oberkochen, Germany) was used to observe the microscopic morphology of the polymer gel before and after 30 d of aging in a CO_2_ environment at 110 °C, a salinity of 1.0 × 10^5^ mg/L, and a pressure of 20 MPa in order to evaluate its long-term CO_2_-resistant performance.

### 3.7. Oil Displacement Experiment

A high-temperature and high-pressure multifunctional core flooding apparatus was employed to evaluate the enhanced oil recovery potential of the smart responsive polymer gel for channel blocking. First, the dehydrated and degassed crude oil and the fractured core were vacuumed, after which the core was pressurized and saturated with crude oil, followed by aging in an oven at 110 °C for 2 d. The fractured core was then mounted in a core holder, and a confining pressure was applied and maintained at least 3.0 MPa higher than the injection pressure throughout the experiment. CO_2_ was injected into the core at a constant flow rate of 0.20 mL min^−1^ to conduct the first CO_2_ flooding stage. Subsequently, a predetermined volume of the gel-forming solution was injected into the core at the same flow rate of 0.20 mL min^−1^. The core holder was then sealed and aged at 110 °C for 48 h to ensure complete gelation of the gel-forming solution. Thereafter, a second CO_2_ flooding was performed by injecting CO_2_ into the core again at a constant flow rate of 0.20 mL min^−1^. Throughout the experiment, the displacement pressure difference as well as the produced gas and liquid volumes at the outlet were continuously recorded. The specific fractured core parameters are provided in Table 1.

## 4. Conclusions

In this work, a smart responsive polymer was synthesized and subsequently crosslinked with a highly active phenolic resin crosslinking agent to develop a smart responsive polymer gel channel blocking system. The gel exhibits CO_2_-responsive strength enhancement and excellent CO_2_-resistant performance. The static and dynamic gelation behaviors, nonlinear rheological properties, CO_2_-resistant performance, channel blocking, and enhanced oil recovery performance of the smart responsive polymer gel were systematically investigated. The results demonstrate that the polymer gel maintains good structural stability during dynamic transport in the reservoir and does not undergo significant strength degradation under shear conditions. Moreover, the smart responsive polymer gel exhibits excellent CO_2_-resistant performance within a temperature range of 80–110 °C, salinity up to 10 × 10^4^ mg/L, and pressure up to 20 MPa. Moreover, the system shows a significant enhancement in channel blocking and enhanced oil recovery performance, highlighting its promising potential for effective CO_2_ flooding channel blocking in low-permeability reservoirs.

It should be noted that the current evaluation was conducted under controlled laboratory conditions. The performance of the gel system in highly heterogeneous or complex fracture networks may be influenced by factors such as fracture connectivity, scale effects, and in situ flow dynamics. Challenges related to field-scale implementation, including gel placement control, injectivity, and long-term stability in complex reservoir architectures, warrant further investigation. Future work should focus on large-scale physical simulations and field trials to validate the applicability and robustness of the proposed system under realistic reservoir conditions. Nevertheless, this study provides a novel technical pathway and a solid theoretical foundation for the application of polymer gels in CO_2_ channeling control and CCUS geological storage.

## Figures and Tables

**Table 1:** Specific fractured core parameters.

Length/cm	Diameter/cm	Permeability/mD	Porosity/%
10.07	2.48	9.65	15.75

## Data Availability

The original contributions presented in this study are included in the article. Further inquiries can be directed to the corresponding authors.
